# Knowledge, Attitudes, Religiosity, and Reproductive Genetic Carrier Screening: A Cross-Sectional Study

**DOI:** 10.3390/healthcare14193316

**Published:** 2026-10-05

**Authors:** Keren Dopelt, Yifat Findling

**Affiliations:** 1 Department of Health Systems Management, Ramat-Gan Academic College, Ramat-Gan 52275, Israel; 2 Department of Nursing, Ashkelon Academic College, Ashkelon 78211, Israel; yifat.f12@gmail.com

**Keywords:** genetic carrier screening, genetic testing, knowledge, attitudes, religiosity, perceived barriers, reproductive health, Israel

## Abstract

**Highlights:**

**What are the main findings?**

- Previous genetic carrier screening was reported by 37% of participants; fear of results and insufficient knowledge were the leading perceived barriers.
- In the fully adjusted model, favorable attitudes and having children at the time of the survey were the only independent correlates of previous genetic carrier screening.

**What are the implications of the main findings?**

- Carrier-screening programs may benefit from combining clear information with emotionally sensitive, culturally responsive, and practically accessible counseling.
- Physicians are a key point of intervention, although consultation preferences should not be interpreted as a direct measure of trust.

**Abstract:**

**Background/Objectives:** Reproductive genetic carrier screening for autosomal recessive and X-linked conditions is an important component of preconception care, yet uptake may be influenced by informational, attitudinal, cultural, and religious factors. Few studies have examined genetic knowledge, attitudes, and religiosity jointly in relation to self-reported carrier-screening behavior. This study characterized previous carrier screening, perceived barriers, decision influences, and preferred consultation sources among adults in Israel and assessed the independent contributions of these psychosocial factors beyond sociodemographic characteristics and reproductive life stage. **Methods:** A cross-sectional online survey was completed by 331 adults aged 18–60 years. The questionnaire introduction defined the study context as genetic carrier screening; subsequent abbreviated references to genetic testing were intended to refer to carrier screening within that frame. Measures assessed self-reported previous carrier screening, genetic knowledge, attitudes, religiosity, perceived barriers, and preferred consultation sources. Descriptive, bivariate, and hierarchical binary logistic regression analyses were conducted. **Results:** Overall, 37% reported previous genetic carrier screening. The leading perceived barriers were fear of results (66%) and insufficient knowledge of carrier screening’s purpose (61%). Physicians were the most frequently reported decision influence (62%) and preferred consultation source (80%). The final combined hierarchical logistic regression model was significant relative to the intercept-only model, χ^2^(10) = 131.25, *p* < 0.001, with a Nagelkerke R^2^ of 0.455. More favorable attitudes were associated with higher odds of previous carrier screening (OR = 4.329, 95% CI [2.369, 7.909]), whereas participants without children at the time of the survey had lower odds than those with children (OR = 0.080, 95% CI [0.031, 0.202]). Knowledge and religiosity were not independently associated with previous carrier screening after adjustment. **Conclusions:** A minority of participants reported having previously undergone genetic carrier screening, despite broad recognition of the importance of preconception or premarital screening. Carrier-screening programs may benefit from addressing emotional, informational, cultural, and practical barriers through proactive, culturally responsive, autonomy-supportive clinician communication in preconception and primary-care settings.

## 1. Introduction

Congenital anomalies and genetic disorders constitute a substantial global public health burden [1]. Globally, congenital disorders are estimated to affect approximately 6% of live births and contribute to approximately 240,000 neonatal deaths each year, making them an important contributor to neonatal and under-five mortality [2]. These global estimates encompass a broad range of congenital and genetic conditions and are not specific to the autosomal recessive and X-linked disorders targeted by reproductive carrier screening. In Israel, the national carrier-screening program underscores the ongoing public health relevance of inherited disorders [3].

Reproductive genetic carrier screening is an important component of preconception and prenatal care that can identify individuals who carry a pathogenic variant associated with an autosomal recessive or X-linked condition, thereby helping identify individuals or couples at increased risk of having an affected child. In this study, the term “carrier” refers to carrier status in this reproductive context and does not refer to presymptomatic carriers of autosomal dominant conditions or to balanced chromosomal rearrangements. Carrier screening may be offered broadly to individuals or couples planning a pregnancy or may be prompted by specific risk factors, such as a family history or known increased population risk. It should be distinguished from diagnostic genetic testing and newborn screening, which serve different clinical purposes. When offered before conception or early in pregnancy, screening can clarify reproductive risks and support informed, values-consistent decision-making regarding available reproductive options [4,5]. Advances in genomic technologies have broadened the range and accessibility of carrier screening, making the offer of carrier screening an increasingly integral component of reproductive care, while the decision to undergo screening remains voluntary and preference-sensitive [6]. Israel’s national carrier-screening program was substantially expanded in late 2024 and now provides a uniform, optional, publicly funded panel covering 650 pathogenic variants in 290 genes [7].

The Israeli population is ethnically, culturally, and religiously diverse, and the prevalence of specific inherited disorders varies across communities [8,9]. Nationally available screening may broaden equitable access to reproductive risk information. Nevertheless, availability alone does not ensure participation [4,7]. Administrative data from Israel indicate that 919,820 individual carrier-screening tests were performed between 2015 and 2017, with the annual number of tests increasing by 14.9% over that period. However, the number of people screened could not be determined because individuals may have undergone multiple condition-specific tests [10]. International research consistently shows a gap between general support for carrier screening and personal uptake. In Belgium, for example, 86% of reproductive-aged adults supported offering reproductive genetic carrier screening to couples planning a pregnancy, whereas only 61% indicated that they would consider undergoing screening themselves [11]. In Israel, relatively low participation has been reported in some minority communities, including the Arab population [12]. This is particularly important in the Israeli context, where rates of consanguineous marriage and the prevalence of some autosomal recessive conditions vary across communities, underscoring the need for culturally and linguistically accessible carrier-screening services [13]. These differing patterns suggest that screening decisions are shaped by interacting healthcare-system, community, and sociocultural factors [14].

Barriers to screening are correspondingly multidimensional. Individuals may fear the emotional consequences of a positive result, as well as stigmatization or discrimination [15,16]. In some communities, concerns may also extend to family reputation, disclosure of hereditary conditions, and marriage prospects [16,17]. Other barriers include low perceived susceptibility, uncertainty about the meaning or usefulness of test results, and limited awareness of carrier screening [11]. Religious or ethical concerns, mistrust arising from ineffective communication, and limited access to linguistically and culturally responsive counseling may create additional obstacles, particularly among ethnocultural minority populations [17]. Healthcare professionals may also function as important facilitators: clear recommendations, understandable information, and accessible counseling can make screening more comprehensible and actionable [14,18].

Knowledge and attitudes are among the most frequently discussed determinants of genetic testing behavior. Across diverse settings, respondents often report favorable views of genetic testing despite limited understanding of inheritance, carrier status, and the implications of screening. Studies from Saudi Arabia, Ecuador, and Europe have similarly reported favorable attitudes toward genetic testing alongside limited or moderate genetic knowledge, with greater genetic literacy associated with more favorable attitudes [19,20,21]. A recent systematic review concluded that limited understanding could impede informed decision-making and highlighted the need for clearer public education and communication about genomic medicine [22].

Two established behavioral frameworks help explain how these factors may relate to screening. The Health Belief Model posits that preventive action is more likely when individuals perceive a meaningful health threat, believe the benefits of action outweigh the barriers, and have adequate cues and knowledge to act [23]. The Theory of Planned Behavior similarly identifies attitudes, perceived social norms, and perceived behavioral control as antecedents of behavioral intention and action [24]. Consistent with these models, greater genetic knowledge has been associated with more favorable attitudes toward testing, while favorable attitudes have been linked to willingness to undergo screening and to screening uptake [11,20,25].

Religiosity may further shape this pathway. Religious and value-based beliefs can influence how individuals understand reproductive responsibility, the acceptable uses of genetic information, and the reproductive options considered following an increased-risk result [17,26]. However, the direction of this influence is not uniform, as associations between religiosity and willingness to undergo genetic testing may vary across national and cultural contexts [27]. Religious norms may discourage testing when it is associated with morally contested reproductive decisions. Conversely, community endorsement may normalize and promote participation. Community-based premarital screening programs such as Dor Yeshorim illustrate how carrier screening can be adapted to, and reinforced by, the values and social structures of Orthodox and ultra-Orthodox Jewish communities [16,28,29].

Although previous studies have identified psychosocial correlates of genetic testing, many have focused on selected populations, examined knowledge or attitudes in isolation, or assessed intentions rather than self-reported previous genetic carrier screening [11,19,25]. Evidence on how knowledge, attitudes, and religiosity jointly relate to self-reported previous genetic carrier screening remains limited, particularly in culturally and religiously diverse populations.

To address this gap, the present study examined genetic knowledge, attitudes toward genetic testing, and religiosity simultaneously in relation to self-reported previous genetic carrier screening among adults in Israel. Unlike studies that focus primarily on screening intentions or examine these psychosocial factors in isolation, the present study assessed their independent contributions after accounting for sociodemographic characteristics and reproductive life stage. We hypothesized that (1) greater genetic knowledge would be associated with more favorable attitudes toward genetic testing; (2) more favorable attitudes would be independently associated with higher odds of self-reported previous genetic carrier screening; and (3) level of religiosity would be independently associated with self-reported previous genetic carrier screening after adjustment for knowledge, attitudes, sociodemographic characteristics, and reproductive life stage. Given the mixed findings in previous research, no directional hypothesis was specified for religiosity.

## 2. Materials and Methods

### 2.1. Study Design, Setting, and Participants

A cross-sectional online survey was conducted among adults residing in Israel. Participants were recruited using convenience sampling supplemented by snowball dissemination. Both authors distributed the survey invitation through WhatsApp Messenger (Version 25.37.76; WhatsApp LLC, Menlo Park, CA, USA) groups to which they belonged, including student groups, and posted it in Facebook (Version 543.0.0; Meta Platforms, Inc., Menlo Park, CA, USA) groups for pregnant women and young parents. They also shared the invitation with colleagues and family members. Group members and personal contacts were asked to forward the invitation to others. Data were collected from 13 December 2025, to 23 April 2026, using a self-administered questionnaire hosted on Qualtrics (Qualtrics, Provo, UT, USA). Participation was voluntary, and no probability-based sampling frame was used. Eligibility criteria included being aged 18 years or older and residing in Israel. A total of 524 survey entries were recorded. An operational inclusion threshold of at least 90% questionnaire completion was applied to ensure sufficient information for scale scoring and multivariable analysis. Religion was also recorded in the survey. Ten respondents identified as Muslim; because this subgroup was too small to support meaningful comparative analysis, these respondents were excluded from all analyses. After applying the completion threshold and this exclusion criterion, 331 respondents constituted the final analytic sample, representing 63.2% of all recorded entries and predominantly reflecting Jewish participants. 

### 2.2. Tools

The closed-ended questionnaire was developed using items adapted from previously published instruments, principally Alotaibi et al. [19]. Items were translated from English into Hebrew and then back-translated into English to assess consistency with the original wording. The questionnaire was administered in Hebrew. No Arabic-language version was available. The Hebrew version was reviewed by a physician with expertise in public health and epidemiology for content relevance and clarity. Comprehensibility was subsequently pilot-tested with five public health students, whose feedback was used to refine the wording and confirm that the questions were understandable before the survey was administered. These procedures assessed linguistic equivalence, content relevance, and comprehensibility but did not constitute a full psychometric validation of the adapted Hebrew measures. The final questionnaire required approximately 10 min to complete and consisted of six sections:

Sociodemographic characteristics. Participants reported sex, age, relationship status, current parenthood status at the time of questionnaire completion, self-identified level of religiosity, and educational attainment. Among the predominantly Jewish analytic sample, religiosity was categorized as secular, traditional, or religious. These categories reflected participants’ self-identification rather than religious affiliation or membership in different religions. Ethnicity was not assessed. Education was grouped as non-academic (secondary or postsecondary vocational), academic degree, or current student.

Previous genetic carrier screening history and future intention. The questionnaire introduction explained that this study concerned genetic carrier screening for inherited conditions. Before completing the questionnaire, participants were provided with brief definitions distinguishing genetic disease from inherited disease. Inherited disease was described as a condition transmitted from parents to children through a pathogenic gene variant, with the additional explanation that both parents may be healthy carriers while their child may be affected; cystic fibrosis was provided as an example. This wording was intended to anchor the concept of carrier status in the context of recessively inherited disease. Within that defined context, the primary behavioral outcome was self-reported previous genetic carrier screening, assessed by asking participants whether they had previously undergone genetic testing (no = 0, yes = 1). Accordingly, subsequent abbreviated references to “genetic testing” were intended to refer to carrier screening within this previously defined reproductive and inherited-disease context. Participants also reported whether either parent had undergone genetic testing and, among those without previous carrier screening, whether they intended to undergo carrier screening in the future. Previous carrier screening and future screening intention were treated as distinct variables. Future intention was assessed only among participants who reported no previous carrier screening and was reported descriptively; it was not combined with previous screening in the regression analyses. Participants could select multiple circumstances in which they believed carrier screening was important: before marriage or pregnancy planning, during pregnancy, only when there is a known genetic risk or family history, or only in the presence of medical symptoms.

Perceived barriers. Respondents selected all factors that they believed could prevent or delay carrier screening. These were self-reported perceived barriers and were not inferred from participants’ knowledge scores or assessed by the researchers. Response options included fear of discovering a problem, perceived insufficient knowledge of the test’s purpose, high cost or lack of insurance coverage, difficulty understanding results, opposition from a partner or family member, religious or cultural reasons, mistrust of the healthcare system, limited time or availability, inconvenience, and an open “other” option.

Decision influences and consultation preferences. Participants selected all factors that influenced their decision to undergo or decline carrier screening, including a physician’s recommendation, a partner’s recommendation, religious or halakhic considerations, social pressure or norms, family history, and an open “other” option. They also indicated whom they would consult about carrier screening: a physician, partner, rabbi or other religious leader, family member, friend, genetic counselor, or online/social-media source.

Attitudes toward genetic testing. Attitudes were assessed with 12 items adapted from Alotaibi et al. [19]. The scale included both personally framed items (e.g., interest in learning about one’s own genetic risks or concerns about one’s own test results) and more general or normative items concerning the perceived implications, availability, and social consequences of genetic testing and carrier screening. Accordingly, the scale reflects overall attitudes toward genetic testing rather than a measure of personal future screening intention. Responses were recorded on a five-point scale ranging from 1 (not at all) to 5 (to a very great extent). Items 5–7, 9, and 10 were reverse-scored so that higher values consistently indicated more favorable attitudes. The overall score was the mean of the available item responses and could range from 1 to 5. Ten participants had one or more missing attitude items; their scores were based on 9–11 completed items. Internal consistency in the present sample was acceptable (Cronbach’s α = 0.71).

Genetic knowledge. Knowledge was assessed with 15 items adapted from Alotaibi et al. [19], covering genes, inheritance, gene–environment interaction, and genetic variation. Brief definitions of genetic disease and inherited disease preceded the items. Participants classified each statement as true, false, or “do not know.” Responses were scored against the answer key (including reverse-keyed items 1, 8, 9, and 10). Correct answers contributed one point; incorrect, “do not know,” and unanswered items contributed zero points. Participants who did not answer any knowledge items (*n* = 7) were assigned a missing total score. Total scores ranged from 0 to 15, with higher scores indicating greater genetic knowledge.

### 2.3. Ethical Considerations

This study was approved by the ethics committee at Ashkelon Academic College before recruitment began. Before accessing the questionnaire, potential participants viewed an information page describing this study’s purpose and the voluntary nature of participation. Submission of questionnaire responses constituted informed consent. No item was mandatory, and respondents could skip questions or discontinue at any time. The questionnaire did not request names, contact details, or other direct identifiers. Platform-generated technical metadata were not used in the analyses and will be removed from any dataset made available for secondary use.

### 2.4. Statistical Analysis

Analyses were conducted using IBM SPSS Statistics, version 31 (IBM Corp., Armonk, NY, USA). Internal consistency of the attitude scale was evaluated using Cronbach’s alpha. Pearson’s correlation was used to examine the association between the continuous knowledge and attitude scores. Spearman’s rank-order correlations were used to examine associations between ordinal religiosity and knowledge and attitudes. Chi-square tests examined associations between sex, relationship status, parenthood, religiosity, education, and parental testing history and prior genetic carrier screening; participants who did not know their parents’ testing history were excluded only from that comparison. Binary logistic regression was used to model previous genetic carrier screening (no = 0, yes = 1). Future screening intention was not included in these regression models. Separate unadjusted models were first examined to assess knowledge and attitudes. A sociodemographic model then included sex, age, relationship status, parenthood, and education, and a separate psychosocial model included religiosity, knowledge, and attitudes. Finally, a hierarchical combined model entered the sociodemographic predictors in Block 1 and religiosity, knowledge, and attitudes in Block 2. Categorical predictors were indicator-coded. Reference categories were men, being in a relationship, having children, non-academic education, and secular identity. Adjusted odds ratios (ORs) with 95% confidence intervals (CIs), likelihood-ratio chi-square tests, and Nagelkerke pseudo-R^2^ values were reported. The incremental contribution of Block 2 was evaluated using the change in model chi-square. Descriptive and bivariate analyses used all available data. Regression analyses used complete cases for each model: the sociodemographic model included all 331 participants, whereas the psychosocial and combined models included 324 participants after 7 cases with missing predictor data were excluded. All tests were two-sided, and statistical significance was set at *p* < 0.05.

## 3. Results

### 3.1. Sample Characteristics

The analytic sample comprised 331 adults aged 18–60 years (M = 31.64, SD = 11.38). Most participants were women (84%), and more than half were aged 18–27 years (54%). Approximately two-thirds were in a relationship (67%), and half had children (50%). Self-identified religiosity was distributed across secular (32%), traditional (32%), and religious (36%) categories. Almost half of the participants held an academic degree (44%), while 30% were current students. Most participants were salaried employees (72%). Full sample characteristics are presented in Table 1.

### 3.2. Previous Genetic Carrier Screening, Future Intentions, and Perceived Timing

Overall, 123 participants (37%) reported previous genetic carrier screening, whereas 208 (63%) had not. Among participants without previous carrier screening, 28% intended to undergo carrier screening in the future, 39% did not intend to, and 33% had not yet decided or considered the issue. Most participants (75%) believed that carrier screening was important before marriage or before planning a pregnancy. Smaller proportions indicated that screening was important only when a known risk or family history was present (27%), during pregnancy (21%), or only after the onset of medical symptoms (8%; Table 2).

### 3.3. Perceived Barriers to Genetic Carrier Screening

The most frequently reported perceived barriers were fear of the results or of discovering a health problem (66%) and self-reported insufficient knowledge regarding the purpose of carrier screening (61%). Perceived financial barriers, including high cost or lack of insurance coverage, were reported by 44% of participants, and 43% selected religious or cultural considerations. Practical barriers related to time, availability, or accessibility were reported by 32%. Less frequently reported barriers included opposition from a partner or family member (22%), mistrust of the healthcare system (20%), and difficulty understanding test results (16%; Table 3).

### 3.4. Decision Influences and Preferred Consultation Sources

A physician’s recommendation was the most frequently reported influence on the decision to undergo or decline carrier screening (62%), followed by family history (41%). Recommendations from a partner (20%) and religious or halakhic considerations (18%) were reported less frequently. When asked whom they would consult about carrier screening, 80% selected a physician. A genetic counselor, partner, and family member were each selected by approximately 38% of participants. Religious leaders (13%), friends (11%), and online or social media sources (9%) were selected less frequently (Table 4).

### 3.5. Genetic Knowledge and Attitudes Toward Genetic Testing

Knowledge scores ranged from 0 to 15 (M = 10.18, SD = 3.52; median = 11, mode = 13). The highest proportions of correct responses concerned the increased genetic risk associated with consanguineous marriage (90%), the combined contribution of genes, environment, and lifestyle to disease (87%), and the distinction between carrier status and having a genetic disease (82%). The lowest proportions of correct responses concerned whether all body cells contain the same genes (30%), whether a gene can be seen with the naked eye (46%), and whether a child of a carrier will always inherit carrier status (56%).

Attitude scores had a mean of 3.51 (SD = 0.57) and an observed range of 1.33–4.75. Most participants strongly endorsed individual autonomy in deciding whether to undergo carrier screening (79%) and access to screening for couples planning a pregnancy (74%). Interest in learning about one’s genetic risks (68%) and agreement that couples planning a pregnancy should consider screening (67%) were also common. At the same time, 49% agreed to a great extent that carrier screening could cause concern among people planning a pregnancy, 27% expressed substantial personal concern that a genetic result could lead to cancellation of a marriage, and 20% believed that screening could increase stigma toward people with genetic conditions.

### 3.6. Associations Among Knowledge, Attitudes, and Religiosity

Knowledge was positively correlated with attitudes toward genetic testing (r = 0.30, *p* < 0.001), indicating that greater knowledge was associated with more favorable attitudes. Religiosity was weakly and negatively correlated with knowledge (r_s_ = −0.12, *p* < 0.05) and moderately and negatively correlated with attitudes (r_s_ = −0.31, *p* < 0.001). Thus, greater religiosity was associated with lower knowledge scores and less favorable attitudes toward genetic testing.

### 3.7. Participant Characteristics Associated with Previous Genetic Carrier Screening

Previous genetic carrier screening differed significantly by sex, relationship status, parenthood, education, and parental testing history (Table 5). Women more frequently reported previous carrier screening than men (40% vs. 22%), and participants in a relationship reported previous carrier screening more often than those who were not in a relationship (48% vs. 16%). Previous carrier screening was also more common among participants with children than among those without children (60% vs. 14%). This comparison relates screening undertaken at any previous time to parenthood status at questionnaire completion; it does not establish whether screening preceded or followed the transition to parenthood. Participants with an academic degree most frequently reported previous carrier screening (51%), compared with those with nonacademic education (28%) and current students (25%). Religiosity was not significantly associated with previous carrier screening, χ^2^(2) = 4.82, *p* = 0.090.

### 3.8. Unadjusted Associations of Knowledge and Attitudes with Previous Genetic Carrier Screening

In separate unadjusted binary logistic regression models, knowledge and attitudes were each associated with previous genetic carrier screening. The knowledge model was statistically significant and yielded a Nagelkerke pseudo-R^2^ of 0.05. Each one-point increase in knowledge score was associated with 12.9% higher odds of having previously undergone genetic carrier screening (B = 0.122, OR = 1.129, *p* = 0.001). The attitude model yielded a Nagelkerke pseudo-R^2^ of 0.14. Each one-point increase in the attitude score was associated with approximately 3.90 times the odds of previous genetic carrier screening (B = 1.360, OR = 3.896, *p* < 0.001). These estimates were unadjusted for sociodemographic characteristics and for the other focal predictor.

### 3.9. Multivariable Logistic Regression Models

Model fit statistics for sociodemographic, psychosocial, and hierarchical logistic regression models are presented in Table 6. In the sociodemographic-only model (N = 331), the predictors were jointly associated with previous genetic carrier screening, χ^2^(6) = 96.61, *p* < 0.001, with a Nagelkerke pseudo-R^2^ of 0.345. Women had higher adjusted odds of previous carrier screening than men (OR = 2.639, 95% CI [1.194, 5.833], *p* = 0.016). Participants without children had lower odds than those with children (OR = 0.101, 95% CI [0.044, 0.231], *p* < 0.001). Education was significant overall (Wald χ^2^(2) = 7.67, *p* = 0.022). Compared with participants with nonacademic education, those with an academic education (OR = 2.398, 95% CI [1.253, 4.590], *p* = 0.008) and current students (OR = 2.476, 95% CI [1.065, 5.758], *p* = 0.035) had higher adjusted odds of previous genetic carrier screening. Age and relationship status were not significant.

The psychosocial-only model, based on 324 complete cases, was also significant, χ^2^_(4)_ = 40.81, *p* < 0.001, with a Nagelkerke pseudo-R^2^ of 0.162. Religiosity was not significant overall (Wald χ^2^_(2)_ = 1.14, *p* = 0.566), and knowledge did not reach statistical significance after adjustment for religiosity and attitudes (OR = 1.072, *p* = 0.083). More favorable attitudes were associated with higher odds of previous genetic carrier screening (OR = 3.614, 95% CI [2.125, 6.147], *p* < 0.001).

A hierarchical combined model was then estimated using 324 complete cases. The sociodemographic block was significant, χ^2^(6) = 92.21, *p* < 0.001, with a Nagelkerke pseudo-R^2^ of 0.338. Adding religiosity, knowledge, and attitudes significantly improved model fit, Δχ^2^(4) = 39.04, *p* < 0.001. The final model was significant, χ^2^(10) = 131.25, *p* < 0.001, and yielded a Nagelkerke pseudo-R^2^ of 0.455.

In the final model, participants without children had substantially lower adjusted odds of previous genetic carrier screening than participants with children (OR = 0.080, 95% CI [0.031, 0.202], *p* < 0.001). Attitudes also remained independently associated with previous carrier screening: each one-point increase in the attitude score was associated with 4.33 times the odds of previous carrier screening (OR = 4.329, 95% CI [2.369, 7.909], *p* < 0.001). Sex, age, relationship status, education, religiosity, and knowledge were not statistically significant after simultaneous adjustment (Table 7).

## 4. Discussion

This study found that 37% of participants reported having previously undergone genetic carrier screening. This study extends previous research by examining reported screening behavior rather than intention alone and by evaluating knowledge, attitudes, and religiosity within a single multivariable framework. Fear of test results and limited understanding of the purpose of carrier screening were the most frequently endorsed barriers, while physicians were the leading influence on carrier-screening decisions and the preferred source of consultation. In the fully adjusted model, favorable attitudes and having children, but not knowledge or religiosity, were independently associated with previous genetic carrier screening. These findings suggest that engagement with carrier screening may reflect not only what individuals know but also how they evaluate its personal relevance and anticipated consequences within their reproductive and social context.

### 4.1. Previous Genetic Carrier Screening, Knowledge, and Attitudes

The contrast between the proportion reporting previous genetic carrier screening and the strong endorsement of preconception or premarital screening is consistent with the frequently observed gap between general approval of reproductive carrier screening and personal action. Van Steijvoort et al. [11], for example, reported high support for offering screening but lower willingness to undergo screening personally. In the present study, among participants without previous genetic carrier screening, fewer than one-third intended to undergo carrier screening, while the remainder either did not intend to do so or remained undecided. These findings suggest that perceiving screening as socially desirable or clinically important does not necessarily translate into action. Moving from favorable views to screening may also depend on perceived personal relevance, willingness to engage with genetic risk information, access to clear information, and the integration of screening into routine healthcare [30,31].

One of the key findings of this study is the distinction between genetic knowledge and attitudes toward genetic testing. Although the two were positively related, they showed different associations with previous carrier-screening behavior. Greater knowledge was associated with more favorable attitudes, supporting the first hypothesis and aligning with studies showing that genetic literacy and attitudes toward testing are positively related [19,27]. Knowledge was associated with prior genetic carrier screening in the unadjusted analysis; however, this association was attenuated after accounting for attitudes and other covariates. In contrast, attitudes remained independently associated with previous carrier screening in both the psychosocial and combined models, supporting the second hypothesis. This pattern is compatible with the Health Belief Model and the Theory of Planned Behavior: factual understanding may contribute to appraisal of risk and benefit, but attitudes may be more proximal to action because they incorporate anticipated consequences, values, and readiness to act [23,24]. The cross-sectional design does not permit mediation or causal interpretation, and previous carrier screening may itself have improved knowledge or attitudes through counseling and experience. Nevertheless, the findings indicate that information-only interventions may be insufficient unless they also address concerns, perceived benefits, and the personal relevance of carrier screening.

### 4.2. Religiosity and Sociodemographic Context

Greater religiosity was associated with lower knowledge and less favorable attitudes, but religiosity was not significantly associated with previous genetic carrier screening in the bivariate comparison or in either adjusted model. Thus, the third, non-directional hypothesis was not supported. This distinction is important. Religiosity may shape beliefs about reproductive responsibility, disclosure, stigma, and acceptable responses to a positive result without uniformly determining whether carrier screening occurs [27]. In Israel, premarital carrier screening has become routinized in parts of the ultra-Orthodox community, particularly among Ashkenazi groups, with rabbinic leadership playing a central role in its normalization [32]. Religiosity should therefore not be treated as a simple barrier or as a proxy for opposition to screening.

Several large unadjusted differences in previous genetic carrier screening were observed by sex, relationship status, parenthood, and education. In the sociodemographic model, sex, parenthood, and education remained significant; however, only parenthood remained independently associated with previous carrier screening in the final combined model. Participants without children had markedly lower adjusted odds of having previously undergone genetic carrier screening than those with children. The association between current parenthood and previous carrier screening is expected, given that reproductive carrier screening is commonly offered during pregnancy planning or pregnancy [3]. Participants who were parents at the time of questionnaire completion would therefore generally have had more opportunities to be offered screening or to undergo screening than those without children. This association should primarily be interpreted as reflecting the reproductive context and opportunities for screening, rather than as a novel finding or evidence that parenthood itself promotes screening uptake. Because parenthood was measured at questionnaire completion and the timing of screening was not recorded, the temporal sequence cannot be established. The attenuation of the other sociodemographic associations in the combined model also suggests overlaps among reproductive stage, education, cultural context, and attitudes. Estimates for men should be interpreted with particular caution because they constituted a small proportion of the sample.

### 4.3. Barriers, Decision Influences, and Practice Implications

The barrier profile helps explain why knowledge alone did not account for previous genetic carrier screening. Fear of discovering a health problem was the most frequently selected barrier, followed by insufficient knowledge of the purpose of screening. Financial concerns, religious or cultural considerations, and constraints related to time or accessibility were also common. Thus, barriers spanned emotional, informational, economic, cultural, and practical domains. These findings are consistent with research emphasizing that informed and autonomous genetic decision-making requires communication that is understandable, culturally responsive, and attentive to the consequences individuals anticipate for themselves and their families [17,22]. The findings also indicate that educational materials should explain inheritance and carrier status. They should also explain how results are interpreted and what reproductive options may follow screening. Information on data protection, costs, and coverage should also be provided [33,34].

Physicians appeared to play a central role in participants’ decision-making: their recommendations were the most commonly reported influence, and 80% of participants selected physicians as a preferred source of consultation. Genetic counselors, partners, and family members were selected less often, although each remained relevant to a substantial minority. Because the questionnaire assessed preferred sources of consultation rather than trust directly, these results should not be interpreted as a validated ranking of institutional or interpersonal trust. Nevertheless, they identify healthcare professionals, particularly physicians, as important points for communication and decision support. Brief, proactive conversations in primary care, obstetric, gynecologic, and preconception settings could clarify eligibility and timing, and address questions and ambivalence. They could also facilitate referral to genetic counseling when more detailed deliberation is needed [31,33,35]. Such communication should remain nondirective and support informed choice rather than presenting screening uptake as the only correct decision [26,33]. Partnerships with religious and community leaders may further improve cultural responsiveness, provided that confidentiality and individual reproductive autonomy are preserved [17,34]. From a health equity perspective, these efforts should be accompanied by attention to financial, linguistic, and digital barriers to accessing screening information and services [36]. More broadly, locally driven public health innovation and social entrepreneurship may help translate community needs into culturally responsive action; however, such initiatives should complement rather than replace systematic governmental planning and accountability [37].

### 4.4. Strengths and Limitations

This study has several strengths. It examined self-reported previous genetic carrier screening rather than intention alone; assessed knowledge, attitudes, religiosity, barriers, and consultation preferences within the same study; and used hierarchical modeling to evaluate whether psychosocial factors added explanatory information beyond sociodemographic characteristics. The inclusion of participants from secular, traditional, and religious groups also enabled a graded examination of religiosity across three categories.

The findings should also be interpreted in light of important limitations. First, the cross-sectional design precludes causal conclusions. Knowledge and attitudes may influence carrier-screening behavior, but screening and counseling may also change them. Second, convenience sampling via WhatsApp v. 25.37.76 and Facebook v. 543.0.0, the online questionnaire, and the exclusion of entries with less than 90% completion may have introduced selection and coverage bias. The sample included disproportionately high percentages of women, young adults, and individuals with academic education or current student status, substantially limiting the generalizability of the findings to the broader Israeli adult population. Accordingly, the findings should not be assumed to represent adults with lower educational attainment or more limited access to digital platforms. In addition, because the small Muslim subgroup was excluded from the analyses, the findings predominantly reflect Jewish participants and should not be generalized to Arab and Muslim communities in Israel. Ethnicity was not assessed, and the questionnaire was not available in Arabic. Therefore, we cannot determine the extent to which Arab or other ethnocultural and linguistic minority populations were represented in the recruited sample. These factors further limit the generalizability of the findings to Israel’s diverse population. Third, although the adapted Hebrew measures underwent translation, content review, and pilot testing, they did not undergo full psychometric validation or factor analysis in the present sample. The attitude scale showed acceptable internal consistency, but the use of available-item means and the treatment of unanswered knowledge items as contributing no correct response may have introduced measurement error. Fourth, the barrier items captured factors that participants believed could prevent or delay screening and should not necessarily be interpreted as barriers personally experienced by each respondent. Finally, although the questionnaire introduction explicitly defined the study context as genetic carrier screening, the primary outcome relied on a single self-report item that used the abbreviated term “genetic testing” and did not record the exact panel, timing, or clinical verification of prior screening. It is therefore possible that some respondents interpreted this item as referring to other forms of genetic testing rather than reproductive carrier screening. Some misclassification, therefore, cannot be ruled out. This study also did not record whether previous carrier screening was population-based or prompted by family history or another specific risk indication; these screening pathways, therefore, could not be distinguished retrospectively. This study also did not assess current pregnancy intentions, prior pregnancies, or contact with fertility services. Importantly, parenthood and relationship status were measured at the time of questionnaire completion rather than when previous carrier screening was considered or undertaken. Therefore, the temporal relationship between these characteristics and previous screening cannot be established.

## 5. Conclusions

Among this sample of adults in Israel, 37% reported having previously undergone genetic carrier screening. More favorable attitudes and having children were the only independent correlates of previous carrier screening in the fully adjusted model, whereas knowledge was associated with screening only before adjustment and religiosity was not independently associated with previous screening. Fear, limited understanding of screening’s purpose, cost, and religious or cultural considerations were commonly perceived as barriers, and physicians were the most frequently reported influence and preferred source of consultation. Because the outcome was a context-framed single self-report item, the findings represent participants’ reports of previous carrier screening rather than verified clinical records. Carrier-screening programs and public health campaigns should move beyond conveying factual genetic knowledge. Interventions should directly target emotional and perceived barriers, such as fears of results, stigma, and uncertainty, by combining clear genetic information with emotionally sensitive, culturally responsive, and autonomy-supportive clinician counseling, enabling individuals and couples to make informed decisions that are consistent with their own values and preferences, including the decision not to undergo screening.

Future studies should use probability-based or quota-based recruitment and intentionally include larger samples of men, ethnocultural and linguistic minority communities, and groups with limited digital access. In particular, adequately powered studies conducted specifically within the Arab population in Israel are needed to examine carrier-screening knowledge, attitudes, perceived barriers, and uptake, as well as the relevance of consanguinity and population-specific patterns of inherited disease. Longitudinal designs beginning before carrier screening would help clarify whether knowledge and attitudes predict subsequent carrier screening and whether clinician counseling changes these factors. Mixed-methods research could clarify how fear, stigma, cost, religious considerations, and family dynamics are experienced across communities, while intervention studies could evaluate the effectiveness of culturally adapted decision aids and proactive clinician communication.

## Figures and Tables

**Table 1:** Sample characteristics (*N* = 331).

Characteristic	Category	*n*	%
Sex	Men	54	16
	Women	277	84
Age group, years	18–27	179	54
	28–37	73	22
	38–60	79	24
In a relationship	Yes	220	67
Has children at the time of the survey	Yes	166	50
Religiosity	Secular	106	32
	Traditional	106	32
	Religious	119	36
Education	Secondary	62	19
	Postsecondary vocational	24	7
	Academic degree	145	44
	Current student	100	30
Employment	Salaried employee	239	72
	Self-employed	17	5
	Not employed	75	23

**Table 2:** Previous carrier screening and future screening intentions.

Measure	Response	*n*	%
Previous genetic carrier screening (*N* = 331)	Yes	123	37
	No	208	63
Future intention among participants without previous carrier screening (*n* = 208)	Intends to undergo carrier screening	59	28
	Does not intend	81	39
	Undecided/not considered	68	33
Parents underwent genetic testing (*N* = 331)	Both parents	35	11
	One parent	18	5
	Neither parent	110	33
	Does not know	168	51
When carrier screening is important *	Before marriage/pregnancy planning	247	75
	During pregnancy	68	21
	Known risk/family history only	90	27
	Only in the presence of medical symptoms	27	8

Note. * Multiple responses were permitted; percentages, therefore, do not sum to 100. The response option “Only in the presence of medical symptoms” did not specify whose medical symptoms were being referred to.

**Table 3:** Reported barriers to genetic carrier screening.

Barrier	*n*	%
Fear of the results or of discovering a problem	217	66
Insufficient knowledge of the test’s purpose	202	61
High cost or lack of insurance coverage	146	44
Religious or cultural reasons	143	43
Limited time, availability, or accessibility	105	32
Opposition from partner or family	74	22
Mistrust of the healthcare system	67	20
Difficulty understanding test results	52	16

Note. Participants could select more than one barrier; percentages, therefore, do not sum to 100.

**Table 4:** Decision-making factors and preferred consultation sources.

Construct	Factor or Source	*n*	%
Decision influence	Physician recommendation	204	62
	Family history	136	41
	Partner recommendation	65	20
	Religious/Halakhic consideration	60	18
	Own knowledge and understanding	41	12
	Social pressure or norms	37	11
Preferred consultation source	Physician	265	80
	Genetic counselor	127	38
	Partner	124	38
	Family	124	38
	Religious leader	43	13
	Friends	35	11
	Internet/social media	30	9

Note. Multiple responses were permitted within each question; percentages, therefore, do not sum to 100.

**Table 5:** Sociodemographic characteristics associated with previous genetic carrier screening.

Characteristic	Category	Previous Carrier Screening, *n*/N (%)	χ^2^ (df)	*p*
Sex	Men Women	12/54 (22) 111/277 (40)	6.17 (1)	0.013
In a relationship	Yes No	105/220 (48) 18/111 (16)	31.37 (1)	<0.001
Has children	Yes No	100/166 (60) 23/165 (14)	75.97 (1)	<0.001
Religiosity	Secular Traditional Religious	48/106 (45) 33/106 (31) 42/119 (35)	4.82 (2)	0.090
Education	Nonacademic Academic degree Current student	24/86 (28) 74/145 (51) 25/100 (25)	21.44 (2)	<0.001
Parents’ testing history †	Both parents One parent Neither parent	16/35 (46) 13/18 (72) 35/110 (32)	11.37 (2)	0.003

Note. † Participants who did not know whether their parents had undergone testing (*n* = 168) were excluded from this comparison. Percentages are row percentages.

**Table 6:** Model fit statistics for the logistic regression analyses.

Model	N	Model χ^2^ (df)	*p*	Δχ^2^ (df)	Nagelkerke R^2^
Sociodemographic	331	96.61 (6)	<0.001	–	0.345
Psychosocial	324	40.81 (4)	<0.001	–	0.162
Hierarchical model: Block 1	324	92.21 (6)	<0.001	–	0.338
Hierarchical model: Block 2	324	131.25 (10)	<0.001	39.04 (4)	0.455

Note. Model χ^2^ values are likelihood-ratio tests against the intercept-only model. For the hierarchical analysis, Δχ^2^ represents the improvement in model fit after religiosity, knowledge, and attitudes were added to the sociodemographic block.

**Table 7:** Final multivariable logistic regression model for previous genetic carrier screening.

Model	Predictor	B	SE	Wald	*p*	OR	95% CI for OR
Final	Women (ref: men)	0.617	0.443	1.940	0.164	1.853	0.778–4.414
	Age	0.001	0.016	0.008	0.930	1.001	0.970–1.034
	Not partnered (ref: partnered)	−0.154	0.402	0.148	0.701	0.857	0.390–1.884
	No children (ref: has children)	−2.532	0.477	28.222	<0.001	0.080	0.031–0.202
	Education overall (df = 2)	—	—	1.444	0.486	—	—
	Academic (ref: nonacademic)	0.403	0.390	1.065	0.302	1.496	0.696–3.214
	Student (ref: nonacademic)	0.516	0.476	1.176	0.278	1.676	0.659–4.261
	Religiosity overall (df = 2)	—	—	1.826	0.401	—	—
	Traditional (ref: secular)	−0.187	0.381	0.240	0.624	0.830	0.393–1.751
	Religious (ref: secular)	0.303	0.388	0.610	0.435	1.354	0.633–2.898
	Knowledge score	0.076	0.050	2.315	0.128	1.079	0.978–1.190
	Attitude score	1.465	0.308	22.703	<0.001	4.329	2.369–7.909

Note. N = 324 complete cases. OR = adjusted odds ratio; CI = confidence interval. The outcome was self-reported previous genetic carrier screening (no = 0, yes = 1). Reference categories are shown in parentheses. Education and religiosity were evaluated as categorical variables; their overall Wald tests are shown before the category-specific contrasts.

## Data Availability

The data presented in this study are available on reasonable request from the corresponding author, subject to approval by the institutional ethics committee. The data are not publicly available due to participant privacy and ethical restrictions. Any dataset provided for secondary use will be de-identified and will exclude platform-generated technical metadata, including IP-address and location fields.
